# Surface Glare Suppression in Retinal Imaging With a Cross‐Polarized Line‐Scanning Color Fundus Camera

**DOI:** 10.1002/jbio.70380

**Published:** 2026-11-05

**Authors:** Xingyu Yang, Zijian Zhang, Xiuju Chen, Philip I. Burgess, Sarah McGhee, Mark Johnson, Ticiana Criddle, Gabriela Czanner, Simon P. Harding, David Wong, Xiaoxin Li, Yalin Zheng, Yaochun Shen

**Affiliations:** ^1^ Department of Electrical Engineering and Electronics University of Liverpool Liverpool UK; ^2^ Department of Eye and Vision Science University of Liverpool Liverpool UK; ^3^ Xiamen Eye Centre, Xiamen University Xiamen China; ^4^ St Paul's Eye Unit Liverpool University Hospitals NHS Foundation Trust Liverpool UK; ^5^ School of Electronics and Computer Science, Faculty of Medicine University of Southampton Southampton UK; ^6^ Liverpool Centre for Cardiovascular Science University of Liverpool and Liverpool Heart & Chest Hospital Liverpool UK

## Abstract

Fundus imaging is critical for the early detection and management of retinal diseases, yet cost and technical limitations remain barriers to broad implementation, especially in resource‐limited settings. Conventional fundus photography and scanning laser ophthalmoscopy (SLO) involve trade‐offs in cost, field of view (FOV), color fidelity, and image contrast, and are often affected by corneal surface glare. We present a novel, low‐cost line‐scanning color fundus camera (LS‐CFC) that integrates both line‐confocal and polarization‐based glare suppression, effectively mitigating corneal surface glare while maintaining high contrast, color accuracy, and a large FOV in nonmydriatic imaging conditions. Using a broadband white LED source, the system avoids the complexity of multilaser alignment and calibration inherent to SLO designs. In clinical tests, the LS‐CFC achieved nonmydriatic imaging with an approximately 60° FOV and demonstrated comparable image quality to a standard 45° fundus camera (Carl Zeiss FF450).

## Graphical abstract

A line‐scanning color fundus camera combines line‐confocal gating and cross‐polarization to suppress corneal and optical surface glare. Broadband white‐light illumination enables true‐color retinal imaging without pupil dilation across an approximately 60° horizontal field of view, using a compact, relatively low‐cost design.

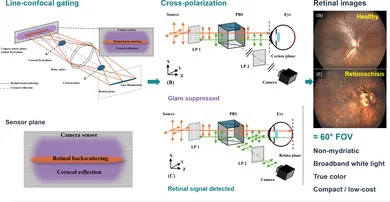

## 1 Introduction

Ophthalmic imaging is an indispensable tool in the documentation and diagnosis of retinal diseases. Digital fundus photography is widely used in diabetic retinopathy screening programs to detect macular oedema and proliferative diabetic retinopathy, the most common causes of vision loss in diabetes [1, 2, 3]. Access to reliable, low‐cost fundus imaging could improve early diagnosis and treatment, reducing preventable vision loss in resource‐limited regions.

Factors such as cost‐effectiveness, artifact suppression, form factor, color, and field of view (FOV) are crucial in designing a fundus imaging instrument. Fundus imaging through the pupil is inherently susceptible to corneal and lenticular reflections, and suppressing these reflections becomes increasingly challenging in wide‐field systems because of the more demanding illumination, collection geometry, and pupil‐utilization requirements. The conventional Gullstrand's Principle‐based approaches require a separation of the illumination and imaging paths for the pupil of the eye to avoid severe reflections from the cornea and crystalline lens [4, 5, 6]. Pupil size remains an important consideration in conventional fundus imaging because it influences both collected signal strength and achievable FOV. Although many contemporary fundus cameras can operate without pharmacological dilation, a smaller pupil can impose greater constraints on illumination and collection geometry, particularly for wide‐field imaging.

Scanning laser ophthalmoscopy (SLO) can provide widefield or ultra‐wide field imaging capabilities in both false and true color formats through confocal rejection of out‐of‐focus and multiply scattered light [7, 8]. However, conventional point‐scanning SLO relies on relatively complex illumination, scanning, relay, and detection optics. Although compact cSLO architectures have shown that this optical complexity can be substantially reduced, achieving a wide FOV while preserving confocality, optical throughput, resolution, and high scanning speeds remains a challenging and highly constrained design problem [7]. Line‐scanning approaches reduce this complexity by illuminating and detecting multiple retinal points simultaneously, although the resulting confocality is partial because scattered light along the line direction cannot be fully rejected [9, 10].

Several approaches have therefore explored the design space between point‐scanning SLO and conventional flood‐illumination fundus photography. Projected‐line and parallel‐line scanning systems have demonstrated confocal retinal imaging with reduced scanning complexity [11, 12]. In parallel, portable and low‐cost fundus cameras have been developed using smartphone‐based imaging, compact indirect illumination and alternative wide‐field illumination geometries [13, 14, 15, 16, 17, 18, 19, 20, 21]. Commercial systems have also continued this trend toward compact and accessible fundus imaging [22]. These developments demonstrate different routes toward more accessible retinal imaging, but trade‐offs remain between optical complexity, confocality, color imaging, FOV and suppression of unwanted reflections.

Cross‐polarization has also been used in fundus imaging to suppress reflections from internal optical surfaces [5]. Other approaches, including miniaturized indirect illumination [16, 17] and trans‐pars‐planar illumination [13, 23] improve pupil utilization or bypass the pupil through contact‐based illumination, enabling portable, nonmydriatic, wide‐field imaging. Further developments have been reported in enhancing dynamic range and improving contrast [16, 19]. However, no single approach fully resolves the competing requirements of wide‐field color imaging, low optical complexity, confocal rejection, and suppression of specular reflections.

We therefore present a nonmydriatic line‐scanning color fundus camera (LS‐CFC) with a 60° FOV that combines line‐confocal gating with cross‐polarization in a compact broadband imaging architecture. The two mechanisms are complementary: line‐confocal gating rejects out‐of‐focus and scattered light, whereas cross‐polarization preferentially suppresses polarization‐preserving specular reflections from the cornea and optical interfaces. Their integration therefore provides broader suppression of unwanted light while retaining a relatively simple scanning architecture. The use of a white light LED source eliminates the need for synthesizing and calibration of multiple lasers, as required in conventional SLOs. We demonstrate the successful implementation of the device with performance characterization and in vivo images obtained from both healthy and pathologic eyes.

## 2 Methods

### 2.1 Optical System Design

The LS‐CFC system is shown schematically in Figure 1A. The light source module contains a visible LED source LS1 (SOLIS‐2C, Thorlabs, United Kingdom) and an infrared (IR) source LS2 with a central wavelength of 780 nm (M780LP1, Thorlabs). Following the definition in the broadband visible source enables true‐color retinal imaging by sampling the continuous spectral reflectance of the retina. The choice of an LED source is not only cost‐effective but also avoids the calibration complexity involved in synthesizing multiple laser wavelengths. A 450 nm long‐pass filter reduces the blue‐light component for improved retinal safety. A ColorChecker (X‐Rite Inc., Grand Rapids, United States) was used to calibrate the camera's response to each color patch.

**FIGURE 1:**
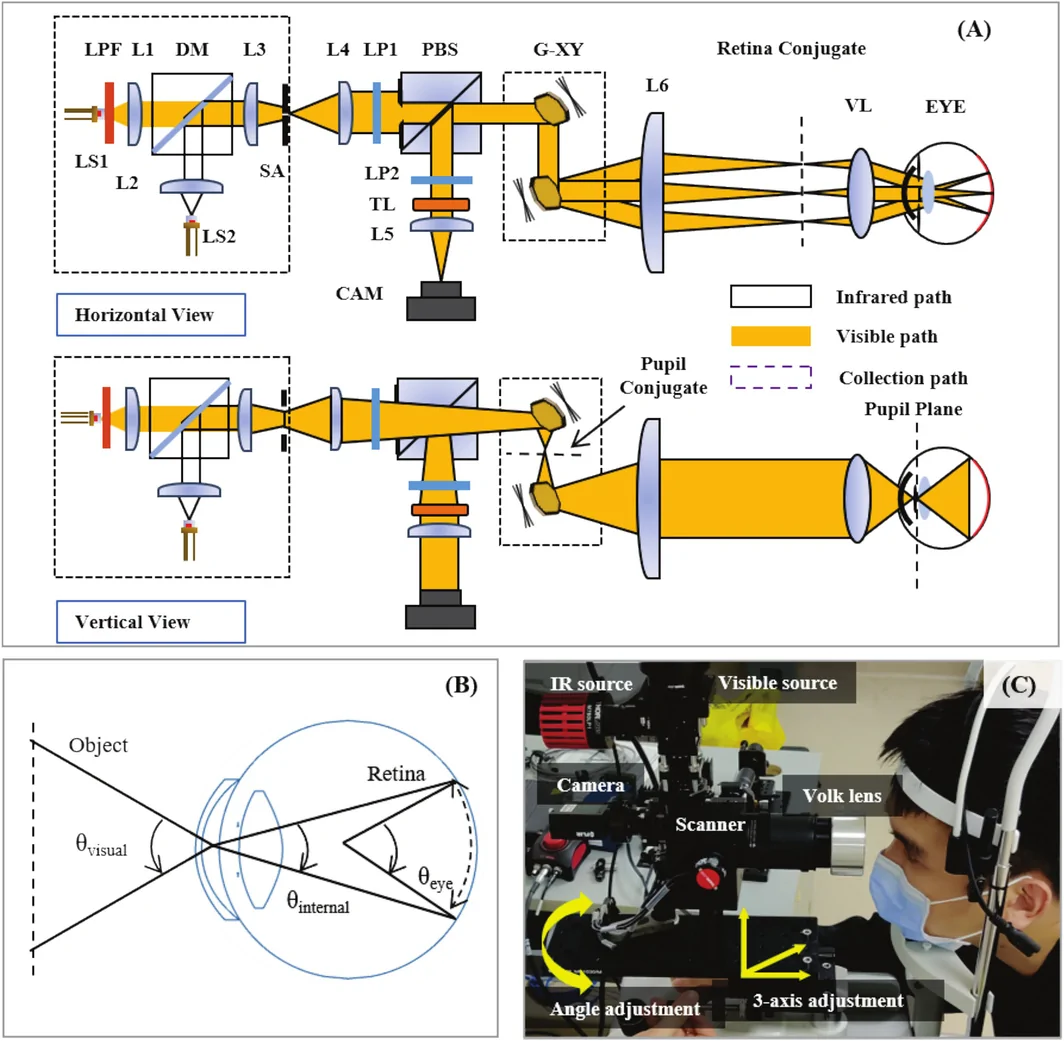
(A) System schematic of the proposed line‐scanning confocal fundus camera shown in vertical (top) and horizontal (bottom) views, illustrating the illumination geometry in the two orthogonal planes. CAM, camera; DM, dichroic mirror; EYE, human eye; G‐XY, dual‐axis Galvo scanner; L1–3, collimators; L4–6, relay lenses; LP1 and LP2, linear polarizers; LPF, long‐pass filter; LS1 and LS2, visible and infrared sources; PBS, polarized beamsplitter; SA, slit aperture; TL, tunable lens; VL, volk lens. (B) External, internal, and eye viewing angles illustrated on a schematic eye. (C) Photograph of the developed system imaging a healthy volunteer.

The IR source used for the measured spectrum at the corneal plane is shown. A short‐pass dichroic mirror DM (DMSP650R, Thorlabs) is used to combine the two sources. The wavelength of the IR source was selected to be distinctly separable from the 840 nm wavelength range to enable potential integration with OCT systems. Light outputs from the two sources are collected by 25 mm focal‐length aspheric condenser lenses L1 and L2 (Thorlabs), and focused to a 120 μm × 10 mm slit aperture SA (Photofab Ltd., United Kingdom) by another condenser lens, L3, with a focal length of 25 mm.

The relay optics follows the principle of indirect ophthalmoscopy and consists of four lenses: a collimating lens L4 with a focal length of 40 mm, a scan lens L6 with a focal length of 80 mm, a focusing lens L5 for the collection path with a focal length of 50 mm, and an ophthalmic lens VL (40D, Volk Optical Inc., USA). A tunable lens TL (EL‐16‐40‐TC, Optotune AG, Switzerland) positioned adjacent to L5 gives an effective focal length of 45 mm and compensates for the chromatic focus shift between IR preview and visible acquisition. An aperture placed before the polarizing beamsplitter PBS (CCM1‐PBS251/M, Thorlabs) limits the illumination beam diameter to match the 10 mm clear aperture of the galvo scanner (GVS012/M, Thorlabs) and prevents beam clipping at the scanner. A two‐dimensional galvo‐scanner (Thorlabs) is placed behind the scan lens L6 for beam steering. Further details of the scanning sequence, strip extraction, correction, and image montaging are provided in the Supporting Materials.

The 120 μm‐wide slit aperture is relayed onto the retinal plane with a paraxial magnification of approximately 1.36, corresponding to a theoretical retinal illumination‐line thickness of approximately 163 μm in a standard emmetropic eye. The pupil plane was identified experimentally as the axial position of maximum illumination intensity, where the illumination beam had a measured 1/e full width of approximately 3.5 mm. This is the diameter of the beam entering the pupil.

The PBS is used to separate the illumination and collection paths. The two linear polarizers, LP1 and LP2 (WP25M‐VIS, Thorlabs) provide additional polarization filtering, as described in Section 2.2. Backscattered retinal light is relayed to a 1920 × 1200 camera (Grasshopper3 GS3‐U3‐23S6C‐C, FLIR Systems UK, United Kingdom) with 5.86 μm pixels. Camera acquisition and galvo scanning are synchronized by a signal output module (NI‐9263, National Instruments Corp, United States). The total system cost is approximately £10 000.

Figure 1B shows the difference in the three viewing angles in a schematic eye: the visual angle *θ*_visual_, the internal angle *θ*_internal_, and the eye angle *θ*_eye_. In this study, the theoretical FOV is calculated using the visual angle *θ*_visual_ [24]. A target visual angle *θ*_visual_ of 60° corresponds to an eye angle *θ*_eye_ of 88.8° and approximately 18 mm of retinal coverage. The 40 D ophthalmic lens and relay optics were therefore designed to support an approximately 60° horizontal FOV through scanning. The primary galvo axis scans the illumination line horizontally across the retina, while the second axis repositions the line vertically between adjacent scan regions. Three vertically offset scans are combined to achieve an approximately 55° vertical FOV. Further details of the scanning sequence are provided in the Supporting Materials. As shown in Figure 1C, the system is installed on a motorized, height‐adjustable slit lamp table by using a customized adaptor that allows horizontal angle adjustment and fine axial adjustment.

### 2.2 Glare Suppression Design

In the LS‐CFC, a combination of line‐confocal and cross‐polarization configurations is employed to suppress specular reflections. The line‐confocal gating of the slit restricts out‐of‐focus light and rejects a substantial portion of reflections from the cornea and internal lens surfaces along the confocal axis, as illustrated in Figure 2A. In this configuration, a slit aperture is used to form a line illumination on the retina, producing an image of the slit at the retinal plane. The backscattered light from the retina is relayed onto the camera sensor, where a defined set of central pixels serves as the detection slit in the collection path. A separate physical detection slit is therefore not required. Multiple sensor rows are retained during acquisition to allow centerline estimation, distortion correction, and adjustment of the effective confocal width before the central pixels are selected for image formation, see Supplementary Material. A conventional single‐line camera would not provide this orthogonal spatial information. The pitch size and number of these pixels determine the effective length and width of the confocal gate. In the present implementation, the central 12 pixels of each strip image, equating to 70.3 μm, are utilized to balance out‐of‐plane light rejection with sufficient signal throughput. However, specular reflections propagating along the line direction continue to contribute significantly to the detected signal.

**FIGURE 2:**
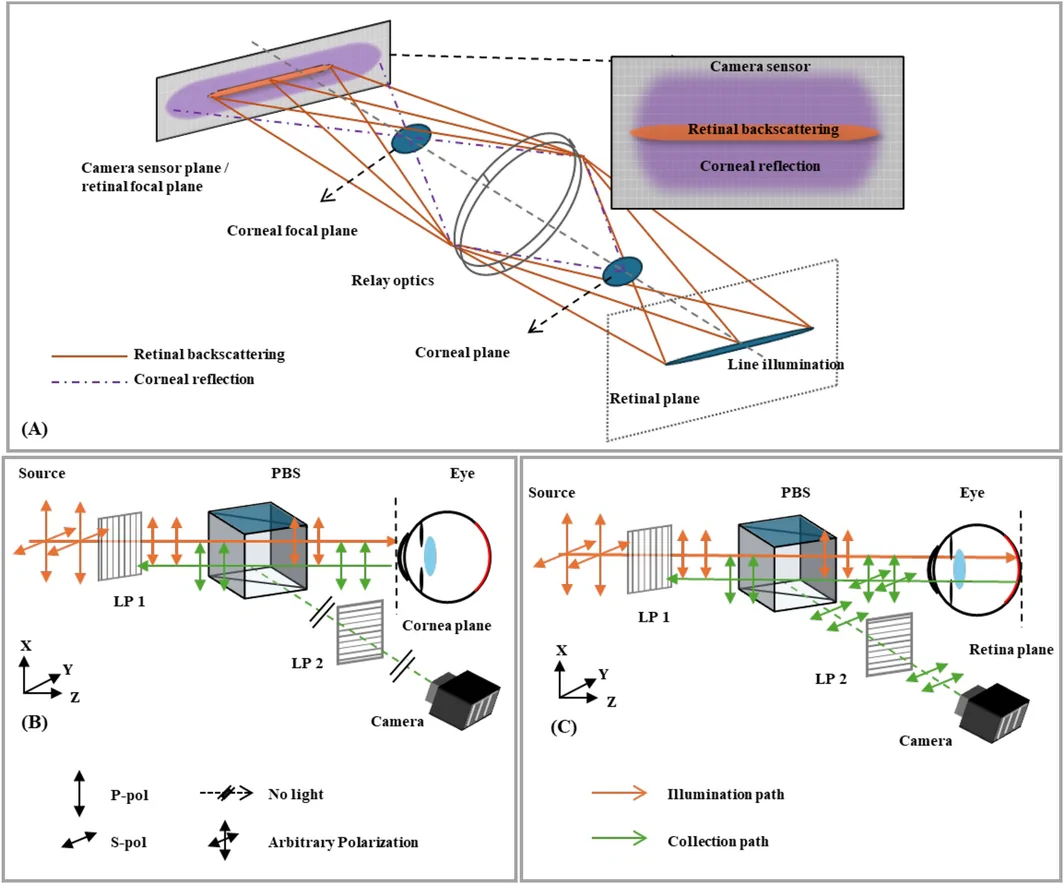
(A) The schematic of the imaging path. The confocal gate formed by the pixels on the sensor largely rejects light reflected from the cornea. Top right corner shows a schematic illustration of confocal gating on the camera sensor plane. CL, camera lens; SL, scan lens; VL, volk lens. (B) The schematics of corneal reflection. The polarization state of the illumination light is preserved. The cross‐polarization configuration blocks the returned light. (C) The schematics of the backscattered retinal signals. The return light is depolarized and can pass through another linear polarizer to reach the camera sensor. LP1 and LP2, linear polarizers; PBS, polarizing beamsplitter. *X*, *Y*, and *Z* correspond to the p‐polarization direction, the s‐polarization direction, and the direction of light propagation, respectively.

To address this limitation, a cross‐polarization configuration is incorporated, as shown in Figure 2B,C. The randomly polarized illumination first passes through LP1, producing p‐polarized light that is transmitted through the beam splitter with minimal energy loss before reaching the retina. The corneal and internal lens surfaces act as polished interfaces that preserve the polarization state of the incident light, whereas light returning from the retinal layers is dominated by multiple‐scattered photons with randomized polarization states (depolarized) [25]. LP2 is placed orthogonal (90°) to LP1, thereby blocking the p‐polarized component reflected from the cornea and lens while transmitting the depolarized backscatter from the retina. Ocular birefringence, diattenuation, and partial depolarization can modify the returning polarization state, with inter‐subject variability also reported [26]. Retinal backscatter therefore contains an orthogonal polarization component that can pass through LP2, while some residual glare may also remain.

PBS provides the primary polarization separation in the system, transmitting the incident p‐polarized illumination and directing the orthogonal return component toward the detector. Because the extinction ratio of the beam splitter for reflection is approximately 10 times worse than for transmission, LP2 provides additional extinction of residual co‐polarized light. The combined line‐confocal and cross‐polarization configuration therefore suppresses rather than completely eliminates corneal and internal optical reflections.

A total of 360 frames are needed to construct a fundus image of 1920 × 2600 pixels. With the camera speed configured to be 1000 frames‐per‐second (fps), the total scan time achieved was approximately 0.4 s. The fundus image produced from our system is constructed by montaging multiple backscattered ‘strip’ images from the retina. Each individual image is acquired by the camera with an exposure time of 1 ms before the galvo scanner shifts to the next position. A detailed discussion of system characterization, image formation, lens distortion compensation, color correction, and optimization can be found in the Supporting Materials.

### 2.3 Practical Considerations

In terms of usability design, a height adjustable slit lamp table is employed for LS‐CFC. The optical components are mounted on a customized adaptor for horizontal and axial fine adjustment. The adaptor is placed on the joystick‐controlled platform for coarse horizontal and axial movement. The ring on the joystick also provides fine adjustment in the vertical direction. A chinrest is installed to position and fix the head of the subject. An external fixation target was attached to the chinrest and used to guide subject fixation during image acquisition.

Positioning and focus are achieved by using the IR light source as the real‐time imaging guide. The IR beam shares the same optical path and image‐forming methods with visible light, including sharing the same single slit as shown in Figure 1A. The illumination focus on the retina is adjusted manually by translating the 40 D ophthalmic lens (VL) along the optical axis, providing refractive‐error compensation of approximately ±6 D in the examined subjects. When the ideal alignment and focusing positions are found, the IR light source is turned off, and the visible light source is turned on for the duration of a full acquisition. The focus position shifts slightly when switching wavelengths because of the human eye's wavelength‐dependent optical properties. The electrically tunable lens is located only in the collection path and therefore does not alter the focus of the illumination beam; it is used to compensate for the wavelength‐dependent focal shift between IR preview and visible acquisition.

### 2.4 Light Safety Considerations

The light safety of the system was evaluated following BS EN 60825‐1:2014 [27] and ISO 15004‐2:2007 [28]. BS EN 60825‐1 specifies maximum permissible exposure (MPE) values for ocular exposure, while ISO 15004‐2 specifies optical radiation safety requirements for ophthalmic instruments. In scanning ophthalmic imaging systems, retinal exposure is reduced by distributing illumination across the scan field, and safety may therefore depend on continued scanning [29]. For a conservative assessment, all calculations assume a worst‐case static‐beam condition, with illumination remaining at a fixed retinal location for the relevant exposure duration.

Optical power was measured at the pupil plane after propagation through the complete illumination path, including the 40 D ophthalmic lens, using a calibrated power meter (S121C, Thorlabs) with a 7 mm limiting aperture positioned at the system working distance. The measured pupil‐plane powers were 1.6 mW for the visible channel and 0.8 mW for the NIR channel. These measurements therefore include the transmission losses introduced by the ophthalmic lens and the preceding illumination optics. The source spectrum was measured using an Ocean Optics USB2000+ spectrometer.

For the visible channel, the retinal thermal MPE was calculated according to in Table A2 of BS EN 60825‐1:2014. The measured angular subtense was 50.8 mrad, giving $C_{6}=\alpha /\alpha_{\min}=50.8/1.5=33.9$. For the 0.4 s acquisition, the MPE was 306.6 J/m^2^, equivalent to an average irradiance of 76.7 mW/cm^2^. The measured visible power corresponds to 4.16 mW/cm^2^, approximately 18.4 times below the MPE.

Retinal exposure was additionally assessed according to the Group 1 limits in Table 2 of ISO 15004‐2: 2007. The illuminated retinal area was measured as 1.2 mm^2^ and the calculated retinal thermal irradiances for the visible and NIR channels were 133.3 and 66.7 mW/cm^2^. Both thermal and photochemical retinal hazards were evaluated using the applicable spectral weighting functions and exposure‐duration limits. The resulting exposure values and safety margins are summarized in Table 1.

**TABLE 1:** Summary of light safety calculations.

Hazard type	Wavelength range	Limit	Measured/calculated	Safety margin
Retinal thermal hazard (MPE)	Visible^a^ (450–700 nm)	76.7 mW/cm^2^	4.16 mW/cm^2^	~18.4× below
Retinal thermal hazard (irradiance)	Visible (450–700 nm)	700 mW/cm^2^	133.3 mW/cm^2^	~5.3× below
	NIR^b^ (700–850 nm)	700 mW/cm^2^	66.7 mw/cm^2^	~10.5× below
Retinal photochemical hazard (duration)	Blue‐light weighted (400–550 nm)	1090 s	0.4 s	2725× shorter than limit

^a^ For the visible channel, the measured spectrum was weighted according to the applicable hazard function, as shown in Figure 3B.

^b^ For the NIR channel, the most conservative wavelength of 700 nm is assumed, despite the light source having a center wavelength of 780 nm with a FWHM of 30 nm.

For the visible channel, the measured spectrum was also weighted using the blue‐light hazard function derived from the ICNIRP guidelines [31], as shown in Figure 3. The resulting blue‐light‐weighted optical power was 0.11 mW, corresponding to a MPE duration of 1090 s. Since the visible source was powered only during the 0.4 s acquisition window, the actual exposure duration was 2725 times shorter than this limit.

**FIGURE 3:**
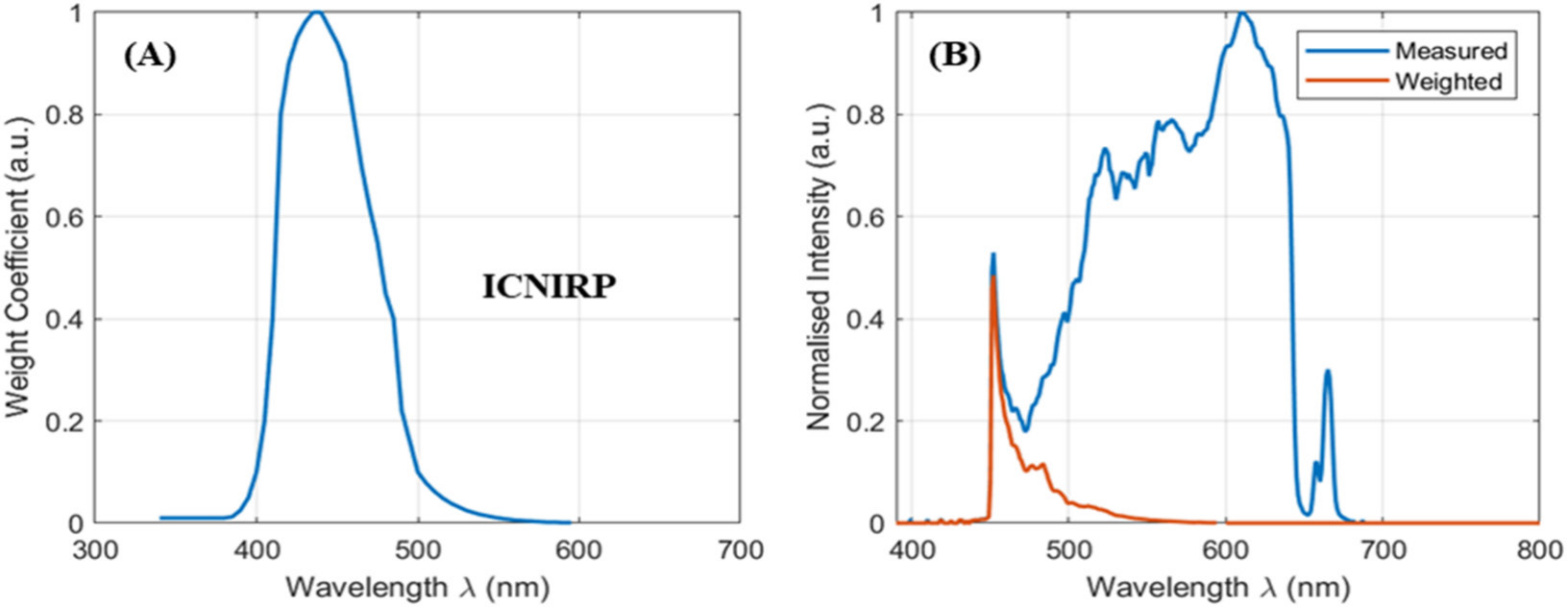
(A) The blue light hazard function derived from ICNIRP [31]. (B) Measured visible‐light spectrum and corresponding hazard‐weighted spectrum.

### 2.5 Human Subjects and Experiment Setup

The clinical study of the prototype was conducted in Xiamen Eye Centre of Xiamen University, China. Eight participants (16 eyes) participated. The healthy participants were aged 25–35 years, while the patients were aged approximately 59–70 years. The image acquisition was conducted in a room with normal light conditions. Fundus image acquisition was performed on 6 of the participants without the use of pharmacological pupil dilation. Two of the participants were imaged before and after applying pharmacological pupil dilation, which consists of 1% tropicamide and 2.5% phenylephrine. Ethical approval was granted by the hospital's institutional review board (case number XMYKZX‐KY‐2022‐036). Written informed consent was obtained from all participants.

### 2.6 Quantitative Image‐Quality Analysis

Quantitative image‐quality analysis was performed on 14 of the 16 enrolled eyes, comprising seven healthy and seven pathological eyes. The remaining two eyes were reserved for the separate pre‐ and postdilation comparison in Figure 5 and were therefore excluded from the cohort‐level analysis. One standard‐protocol LS‐CFC image was included per analyzed eye. One standard‐protocol LS‐CFC image was included per eye. Each eye was analyzed separately, including bilateral images where available. Eight vessel‐crossing line regions of interest (ROIs) were automatically identified from the green channel and manually reviewed or adjusted. Normalized local vessel contrast was calculated as follows:

$$C_{\text{vessel}}=\frac{I_{\mathrm{bg}}-I_{\text{vessel}}}{I_{\mathrm{bg}}},$$

where $I_{\mathrm{bg}}$ was the background intensity defined as the mean of the median intensities within the outer 20% at each end of the profile. $I_{\text{vessel}}$ was the vessel intensity defined as the 20th‐percentile intensity within the central 40% of the profile. The eight measurements were averaged to obtain one mean vessel‐contrast value per eye, with the corresponding SD describing variation among retinal locations within that eye.

Global percentile contrast was calculated within the automatically segmented retinal field. After averaging the red, green, and blue channels, the 10th and 90th percentile intensities, $P_{10}$ and $P_{90}$, were determined, and global contrast was calculated as

$$C_{\text{global}}=\frac{P_{90}-P_{10}}{P_{90}+P_{10}},$$

The group comparisons were descriptive because of the small cohort and the inclusion of bilateral eyes from some participants.

## 3 Results

Figure 4 shows representative fundus images captured by the developed device. The LS‐CFC achieved an experimentally measured FOV of approximately 55° vertically and 60° horizontally, as quantified using an emmetropic model eye. In vivo, optic disc size was used only as an approximate anatomical reference; a typical optic disc diameter is about 1.8 mm, corresponding to a 6° FOV [32, 33]. Retinal magnification varies between individuals and can be estimated more accurately using subject‐specific ocular biometry, particularly axial length [34]. As axial length was not recorded in the present study, the optic‐disc estimate was therefore used only to illustrate approximate retinal coverage. On this basis, the image in Figure 4A spans approximately 10–11 disc diameters horizontally and 8–9 disc diameters vertically. Additionally, in Figure 4B, a standard 45° fundus photograph acquired by a Carl Zeiss FF450 fundus camera is overlaid on an LS‐CFC image to illustrate relative retinal coverage. Collectively, the images produced by the LS‐CFC achieve the intended FOV coverage.

**FIGURE 4:**
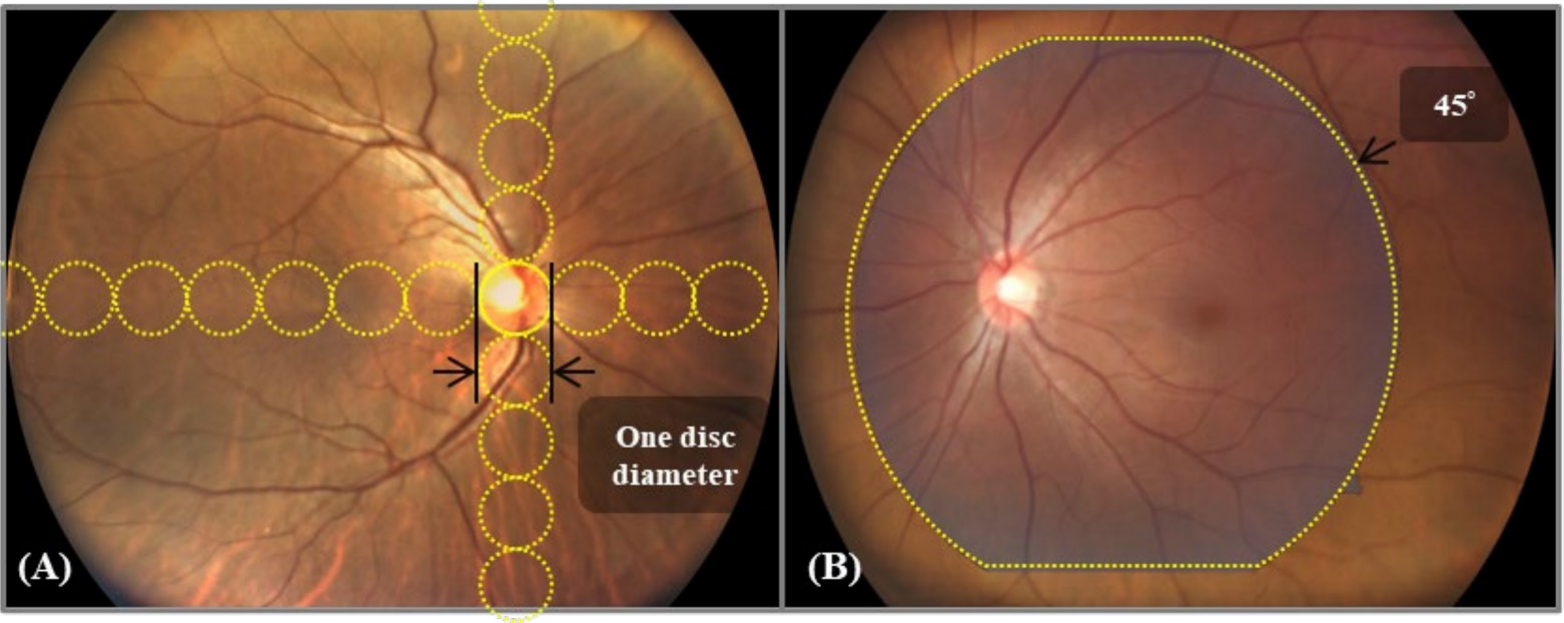
(A) Self‐referencing FOV quantification with optic disc diameter on a fundus image obtained using LS‐CFC. (B) A 45° standard fundus photograph imaged by Carl Zeiss FF450 enclosed by the yellow dashed line is overlayed on a fundus image obtained by LS‐CFC.

The optical resolution of fundus imaging is fundamentally limited by the pupil, which serves as the system aperture. A smaller, undilated pupil restricts both light throughput and spatial resolution. To assess the nonmydriatic imaging performance of the LS‐CFC, fundus images of the same eye were obtained before and after pupil dilation. Figure 5A,F shows the respective images, both achieving comparable FOV. The red and purple rectangles mark the superior and inferior edges of the images, highlighting consistent image quality under both conditions, as further illustrated in Figure 5B–E,G–J for the undilated and dilated pupils, respectively. The optic disc region, indicated by the yellow rectangles, exhibits slightly higher saturation in the postdilation image, whereas fine retinal structures remain clearly resolved in both cases. Figure 5K–N presents line‐intensity profiles along identical vessel regions (red and yellow dashed lines) for quantitative comparison between pre‐ and postdilation conditions. Arrows denote vessel locations. The low‐frequency component was removed to eliminate baseline trends in the profiles; consequently, the plotted values represent relative intensity deviations from the baseline and may take negative values. Blood vessels, which scatter less light, appear as intensity minima in the profiles. Comparable vessel contrast and count under both conditions confirm that the LS‐CFC achieves comparable‐quality imaging without pharmacologic dilation. Overall, the undilated image demonstrates negligible compromises in image quality or FOV.

**FIGURE 5:**
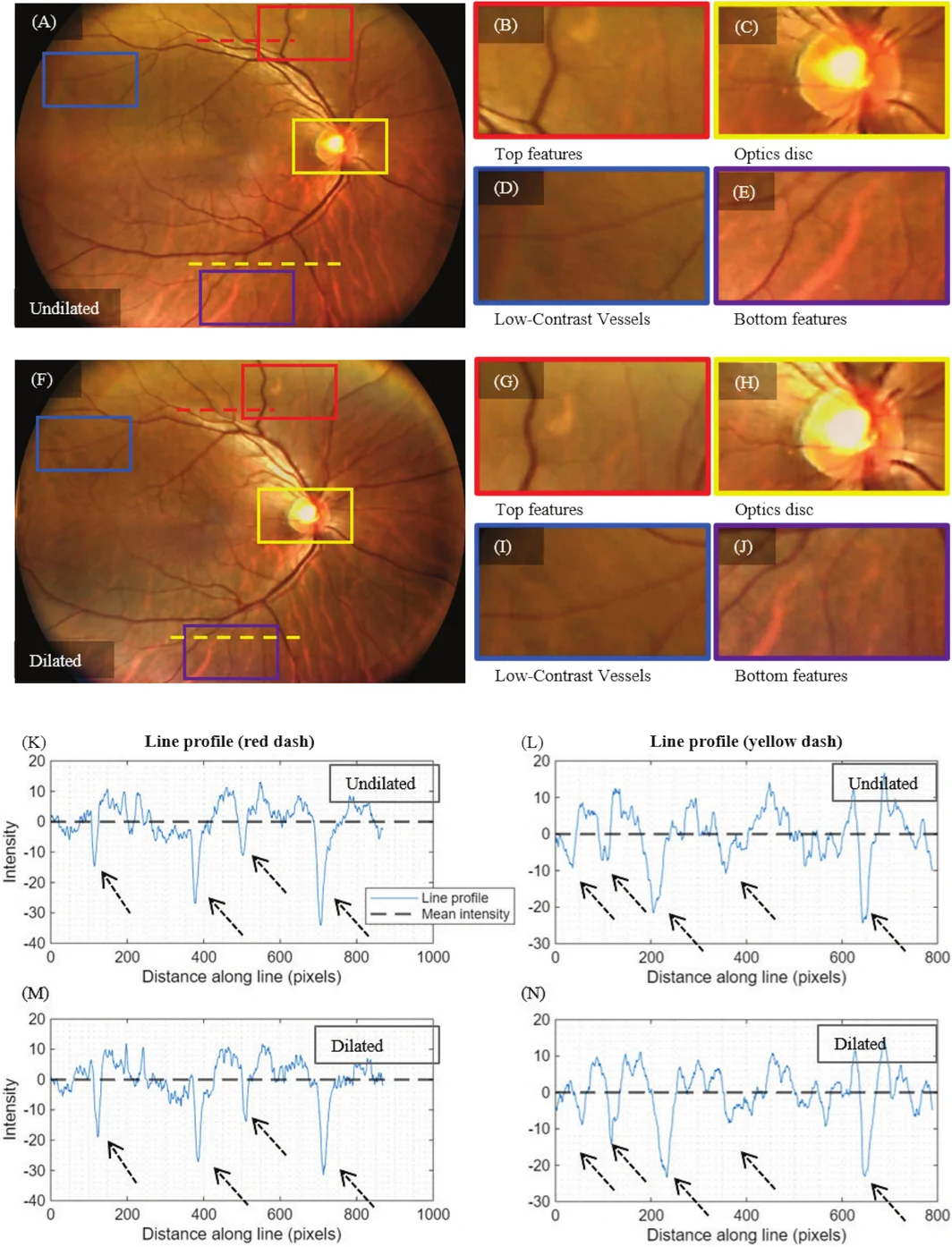
Fundus images of the same subject acquired with the LS‐CFC before (A) and after (F) pupil dilation. Panels (B–E) and (G–J) display color‐coded magnified regions corresponding to the areas marked in the original images. Panels (K–N) show line intensity profiles along the identical vessel regions indicated by the red and yellow dashed lines, comparing pre‐ and postdilation conditions. Arrows are pointed at the locations of blood vessels.

The imaging performance of the LS‐CFC was validated through comparison with a commercial 45° FOV fundus camera that conforms to the Early Treatment Diabetic Retinopathy Study (ETDRS) standard. As shown in Figure 6A,F, images of the same healthy volunteer's eye were acquired consecutively using the LS‐CFC and the Carl Zeiss FF450, respectively. Both the full‐field images and their corresponding magnified regions are presented in Figure 6B–E,G–J. The red and yellow rectangles delineate the superior and inferior edges of the fundus, while Figure 6K–N shows line‐intensity profiles extracted along identical vessel regions.

**FIGURE 6:**
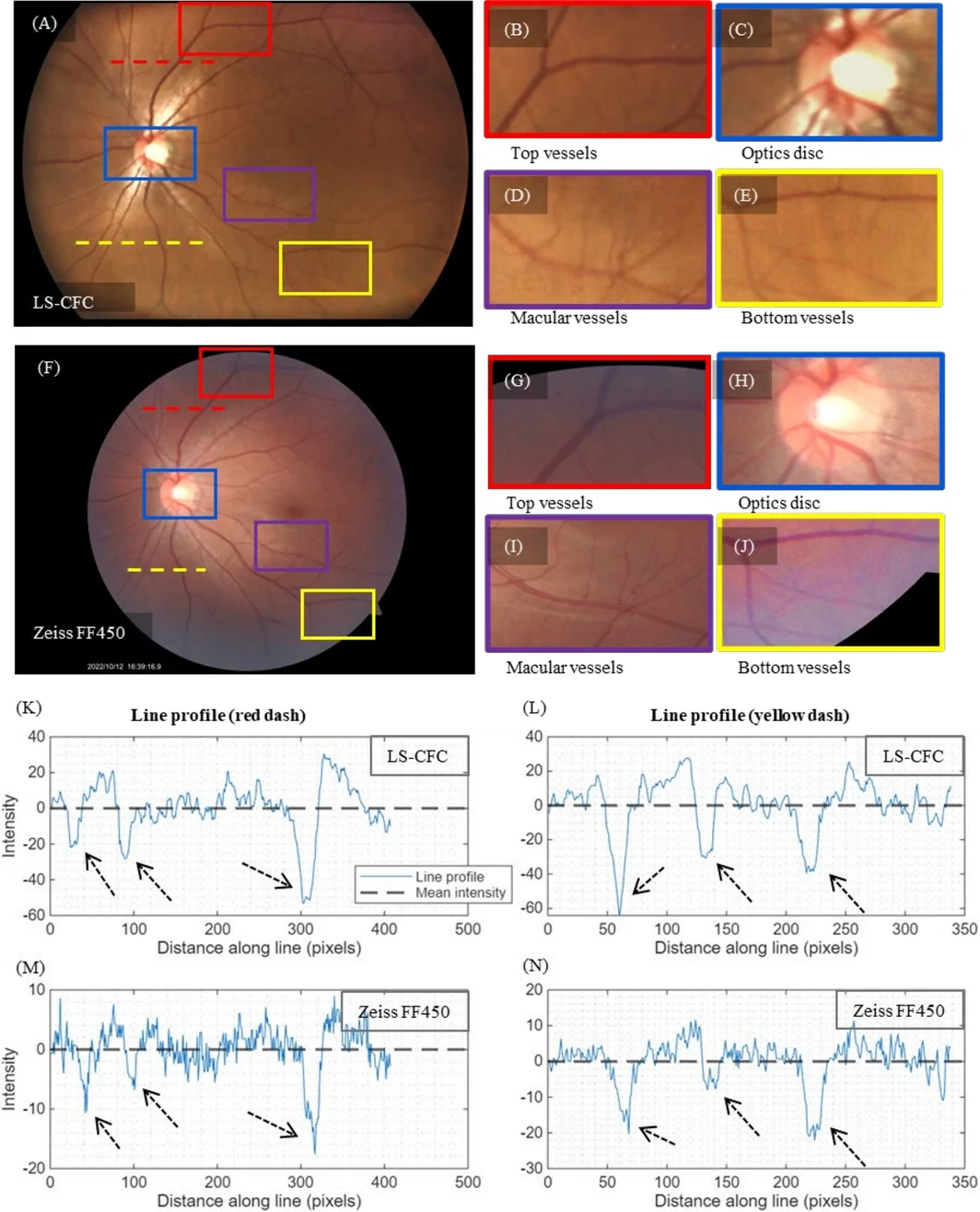
Fundus images of the same subject acquired with the LS‐CFC (A) and the Zeiss FF450 (F). Panels (B–E) and (G–J) display color‐coded magnified regions corresponding to the areas marked in the original images. Panels (K–N) show line intensity profiles along identical vessel regions indicated by the red and yellow dashed lines, comparing image contrast between the two systems. Arrows are pointed at the locations of blood vessels.

Compared with the FF450, the LS‐CFC images exhibit larger FOV, comparable vessel‐to‐background contrast, and improved color uniformity across the field. In contrast, the blue and purple regions in the FF450 images reveal finer structural details and higher local contrast. The reduced contrast in certain LS‐CFC regions is primarily attributed to focus sensitivity; the current optical design employs a shared confocal gate for both illumination and detection paths, resulting in a relatively narrow depth of focus (DoF). As the depth of the optic disc to the rest of the retinal layer ranges from 300 to 700 μm, the out‐of‐focus effect of the disc can be significant if the DoF of the imaging device is insufficient [34].

The ability of the camera to visualize eyes with various pathologies is demonstrated in Figure 7. The figure presents images from three healthy eyes and three pathological eyes, each of which maintains consistent performance in terms of FOV, color, and imaging detail. The ametropia compensation of participants was within ±6D. The three images in the first row were acquired from healthy volunteers with varying degrees of myopia, while images in the second row were from patients diagnosed with macular degeneration, vitreous haze, and retinoschisis, listed from left to right, respectively.

**FIGURE 7:**
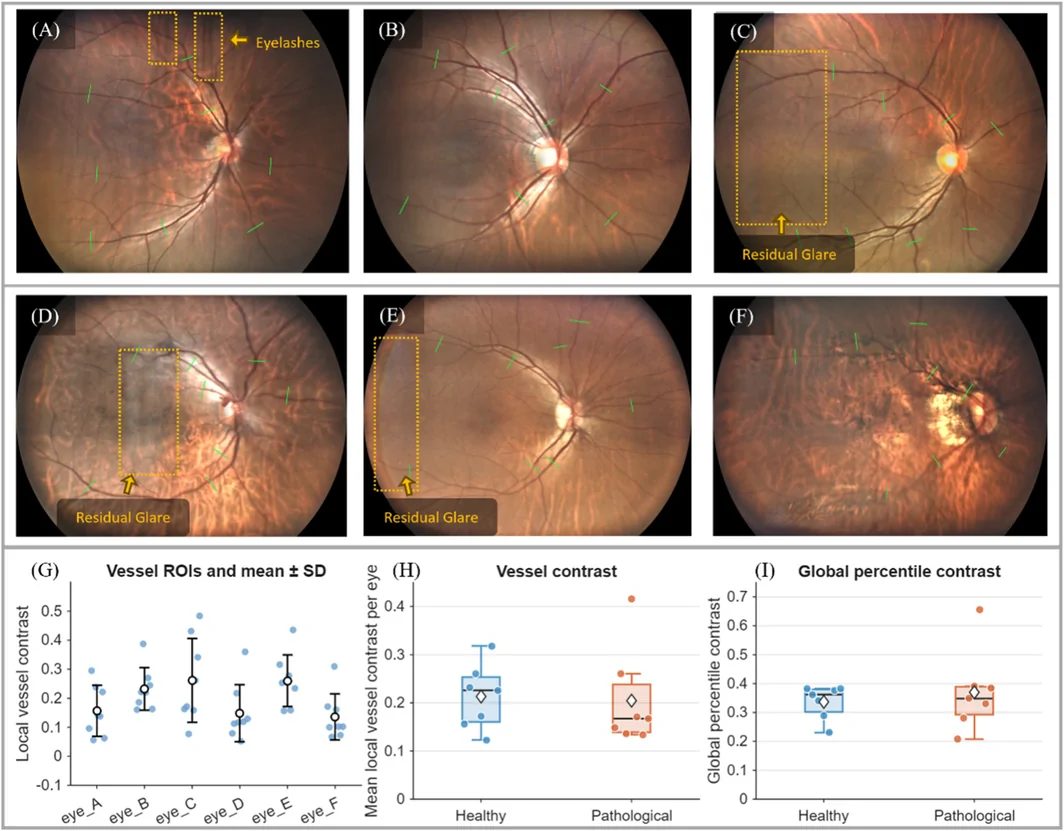
Representative fundus images and quantitative image‐quality assessment obtained using the LS‐CFC. (A–C) Healthy eyes. (D) Macular degeneration. (E) Vitreous haze. (F) Retinoschisis. Green markers indicate vessel ROIs; residual glare is highlighted in (C–E), and eyelash artifacts in (A). (G) Local vessel‐contrast measurements for the six illustrated eyes, with eye‐level mean ± SD. (H) Mean local vessel contrast and (I) global percentile contrast in healthy and pathological eyes (seven eyes per group). Points represent individual eyes, diamonds indicate means, horizontal lines indicate medians, boxes indicate interquartile ranges, and whiskers extend to 1.5 times the interquartile range.

To assess image quality beyond the representative examples, local vessel contrast was measured at eight retinal vessel regions per eye. The vessel ROIs for the six illustrated eyes are indicated by the green markers in Figure 7A–F, and their individual measurements and eye‐level mean ± SD are shown in Figure 7G. The across‐eye analysis included 14 unique eyes: seven healthy and seven pathological eyes. Mean local vessel contrast was 0.213 ± 0.067 in healthy eyes and 0.205 ± 0.102 in pathological eyes, with ranges of 0.123–0.318 and 0.134–0.415, respectively. The distributions overlapped substantially, although the pathological group showed greater inter‐eye variability (Figure 7H). Global percentile contrast was 0.337 ± 0.057 in healthy eyes and 0.371 ± 0.140 in pathological eyes, with ranges of 0.230–0.382 and 0.208–0.655, respectively (Figure 7I). The larger mean and variability in the pathological group were influenced by an eye with particularly high global contrast and should not be interpreted as superior image quality. Collectively, the results demonstrate measurable vessel‐to‐background contrast across all analyzed eyes and broadly maintained local vessel visibility in both groups, while also illustrating the greater image heterogeneity encountered in pathological eyes.

Residual artifacts were also present. Figure 7A contains eyelash artifacts, while the bright regions in Figure 7C–E are consistent with residual specular glare arising from polarization changes in the eye and imperfect polarization extinction. As a general observation, signal intensity from the optic nerve head was also substantially greater than that from the macular region due to the difference in the density of pigmented cells and nerve fibers [35].

## 4 Discussion

### 4.1 Design Rationale and Intended Scope

The developed LS‐CFC system combines line‐scan quasi‐confocal gating with cross‐polarization to enable wide‐field, nonmydriatic retinal imaging in a compact and relatively low‐cost design. Rather than introducing a new polarization principle, the contribution of this work lies in the integration of cross‐polarization within a line‐scan confocal architecture and its application to wide‐field retinal imaging. To the best of our knowledge, this is the first demonstration of such a combined configuration implemented in a compact system designed for large FOV imaging. A brief study has described adopting the cross‐polarization configuration in nonscanning fundus camera imaging of mouse eyes, albeit with a limited FOV [36].

In conventional confocal SLO, the entire pupil is used for both illumination and light collection. Because the conjugate plane of the corneal surface lies well out of focus relative to the confocal pinhole, reflections from the cornea are largely rejected. On the other hand, LSLO employs beam‐displacement optics to spatially separate the illumination and detection paths, thereby reducing corneal reflections. Conventional fundus cameras achieve a similar effect through alternative means, such as using an annular illumination pattern, inserting a light‐absorbing dot at the pupil conjugate plane [3, 6, 37, 38], or optically separating the illumination and collection paths [39, 40]. An annular illumination configuration was evaluated during an earlier design iteration of the system but was not retained because its overall performance was inferior in the present line‐scanning architecture. Introducing an annulus would fundamentally change the optical architecture and reintroduce the geometrical pupil constraints that the LS‐CFC was designed to avoid. The final design therefore combines line‐confocal gating with cross‐polarization to suppress corneal and lens reflections without relying on annular illumination or pupil‐plane separation of the illumination and detection paths.

This study focuses on demonstrating the feasibility, safety, and relative optical efficiency of the LS‐CFC design, rather than comprehensive performance optimization or benchmarking against high‐end true color systems, such as the iCare Eidon or Carl Zeiss Clarus 700, but rather to deliver wider imaging FOV than conventional retinal screening cameras, such as Carl Zeiss FF450 while achieving effective specular glare reduction within a compact and low‐cost platform. We acknowledge the modern ultra‐widefield SLO systems, such as Optis, can provide retinal coverage of up to 200° [41]. However, these systems are less directly comparable because they use discrete laser wavelengths and typically generate false‐color images, whereas the LS‐CFC uses broadband white‐light illumination for true‐color imaging. LS‐CFC therefore occupies an intermediate design space between conventional fundus cameras and more complex scanning modalities. This balance of image quality, system simplicity, and cost makes the approach well suited to applications prioritizing wide FOV, compact footprint, and accessibility.

### 4.2 Imaging Performance

The choice of PBS rather than a nonpolarizing BS has important implications for optical efficiency. In an equivalent 50:50 BS configuration and two linear polarizers, approximately 50% of the returning retinal signal is lost at the beamsplitter and a further 50% of an ideally depolarized return is rejected by the crossed analyzer, giving an effective collection efficiency of approximately 25%. In the present PBS configuration, the orthogonally polarized retinal return is preferentially directed toward the detector, while LP2 primarily provides additional extinction of residual co‐polarized light. The idealized collection efficiency is therefore approximately 50%, about twice that of the equivalent nonpolarizing beamsplitter configuration. This improves retinal signal collection while retaining polarization‐based glare suppression. However, although the overall optical efficiency remains lower than that of split‐path or beam‐stop methods because of the 50% polarization conversion loss, our method is simpler to implement and more readily supports a wider FOV.

An additional advantage of the LS‐CFC architecture is that its achievable FOV is less constrained by pupil geometry than in conventional fundus cameras based on Gullstrand's principle [5, 16]. In the LS‐CFC, the pupil‐conjugate plane is aligned with the center between the *x*‐ and *y*‐galvo mirrors to minimize beam wobble at the pupil during two‐dimensional scanning. Because the two galvo mirrors are spatially separated, perfect pupil conjugation cannot be achieved simultaneously for both scanning axes, resulting in a small residual beam displacement at the pupil. This displacement increases with scan angle and can lead to partial clipping at the pupil edge, particularly for smaller pupils or larger beam diameters. This consideration may be particularly relevant in older populations, as mesopic pupil diameter has been reported to decrease with age [42].

A qualitative comparison between LS‐CFC and related retinal imaging modalities is provided in Table 2 to illustrate the relative mechanisms of glare suppression, optical complexity, and FOV scalability. Commercial devices are not included in this comparison, as the intent is to contrast underlying optical principles rather than specific product implementations.

**TABLE 2:** A comparison of glare suppression methods in fundus imaging techniques.

	Method	FOV (visual)	Confocality	Scanning‐based	Relative cost^a^	Color	Glare artifacts	Contact required
LS‐CFC	Slit + polarization	60°	Quasi	Yes	Low	Multi	Reduced	No
cSLO [8, 30]	Pinhole	Variable	Yes	Yes	High	Mono/False	No	No
LSLO [9, 10]	Slit	Variable	Quasi	Yes	Low	Mono	Reduced	No
Tran et al. [5]	Annulus	50°	No	No	Low	Multi	Yes	No
Fundus Camera [3]	Annulus + black dot	30°	No	No	N/A	Multi	N/A	No
Toslak et al. [13]	Trans‐pars‐planar illumination	150°	No	No	Low	Multi	Yes	Yes
Rossi et al. [16]	Indirect illumination	67°	No	No	Low	Multi	No	No

^a^ Cost is a qualitative relative comparison based on reported system architecture and component complexity rather than commercial purchase price. Where cost or glare performance was not reported in the cited study, this is indicated accordingly.

### 4.3 Low‐Cost Considerations

Cost‐conscious efforts were made throughout the design and construction of the device. Firstly, only the off‐the‐shelf optics were used to construct the system. Custom‐made optics are discussed in [8, 43] for SLO designs to obtain the desired performance. Secondly, rather than using a combination of a slow scanner and a more expensive fast resonator for a raster scan, our system employs two slow scanners because of its line illumination configuration. Thirdly, LED sources were used for both previewing and acquisition, and an area‐scan camera was selected to provide the multiple sensor rows required for image correction and adjustable confocal gating. Although multiline line‐scan cameras are available, the present acquisition is primarily limited by exposure time and available illumination rather than sensor readout speed. This choice also helps maintain relatively low component cost. Mechanically, the system is mounted on a commercial slit lamp table with a customized adaptor, reducing the need for bespoke hardware. Lastly, the system is mounted on a commercial slit lamp table with a customized adaptor, which significantly reduces the need for customization.

With an estimated cost of £10 000, the LS‐CFC occupies a middle ground between scanning‐based and area‐based fundus cameras in terms of both affordability and imaging performance. This estimate reflects the one‐off cost of optical and electronic components used to construct the experimental prototype and does not include engineering development, manufacturing, or regulatory costs associated with commercial deployment. In practice, component costs may decrease with scaled production. Although an inherent trade‐off between cost and performance remains, the proposed configuration offers a relatively cost‐efficient approach that may facilitate wider deployment of retinal imaging systems, particularly in resource‐limited settings.

### 4.4 Limitations and Future Improvements

Although the imaging capability of the device has been demonstrated in a clinical environment, a few aspects can be improved. Currently, the high‐contrast area of the optic nerve and surrounding trunk vessels are used as a visual aid for focusing. Most images obtained in the clinical study were adequately focused, with a small percentage exception. This was likely due to individual variations in media opacity. This can be improved by implementing an improved focus mechanism and increasing the DoF. Second, the present prototype uses an external fixation target. An internal fixation target could be incorporated in future iterations to improve operator convenience and fixation control, particularly for subjects with limited vision.

The full pupil area can be used as the observation window in this device rather than the techniques based on Gullstrand's Principle, where only the central area directly contributes to imaging. Therefore, the FOV and image pixel density can be further improved by adopting a different front ophthalmic lens and a more sensitive imaging sensor with a finer pixel pitch. A higher‐speed camera will enable the integration of high‐dynamic‐range imaging, as reported in [16]. More precise and automated refractive‐error compensation could be achieved by introducing a tunable focusing element into the illumination path. In the present compact configuration, however, the available space between the PBS and galvo scanner is limited. The flexible system design allows for further optimization to meet specific performance requirements. In addition, the scanning‐based design facilitates straightforward integration with other imaging modalities, such as optical coherence tomography (OCT).

## 5 Conclusions

We present the development, validation, and clinical evaluation of the LS‐CFC incorporating a novel glare‐suppression mechanism. The system design and methods for achieving widefield imaging under nonmydriatic conditions are described in detail. The combined use of line‐confocal gating and cross‐polarization substantially suppresses artifacts arising from internal optical reflections and corneal surface glare. A broadband white‐light source is employed to achieve true color imaging with optimized acquisition speed and contrast. This imaging format produces a visual appearance comparable to that of conventional color fundus photography, rather than relying on synthetic reconstruction of multiple wavelengths for pseudo‐color imaging. Preliminary clinical testing on both healthy and pathological eyes demonstrates the imaging performance of the system. The low‐cost, scanning‐based design also facilitates potential integration with OCT systems, providing a foundation for future multimodal retinal screening devices.

## Funding

This research was supported by the Global Challenges Research Fund through the UK Engineering and Physical Sciences Research Council (EP/R014094/1). For the purpose of Open Access, the author has applied a Creative Commons Attribution (CC‐BY) license to any Author Accepted Manuscript version arising.

## Conflicts of Interest

The authors declare no conflicts of interest.

## Supporting information


**Figure S1:** The relationship map for the design constraints and trade‐offs.
**Figure S2:** (A) The model eye used for FOV quantification follows the eye angle. (B) The acquired artificial fundus image of the model eye. The fourth ring represents an eye angle of 80°, which is a close approximation of a 60° external angle. (C) The quantified system resolution with a central‐installed USAF 1951 resolution target.
**Figure S3:** Image formation and correction for line‐shaped illumination. (A) Upsampled strip image showing the detected centerline, polynomial fit, and corrected result. (B) Schematic of strip extraction, realignment, and montage. (C) Normalized red, green, and blue intensity distributions along the line direction.

## Data Availability

The data that support the findings of this study are available from the corresponding author upon reasonable request.
