# Supplementary material for: Surface Glare Suppression in Retinal Imaging With a Cross‐Polarized Line‐Scanning Color Fundus Camera
Source: J Biophotonics. 2026 Nov 5;19(11):e70380. doi: 10.1002/jbio.70380 (PMC13739417; doi:10.1002/jbio.70380)
Supplement: Supplementary file 1 [file JBIO-19-e70380-s001.docx]

Supporting Materials

S1: Design constraints

**Fig. S1.** The relationship map for the design constraints and trade-offs.

The system is primarily constrained by retinal signal strength, eye safety limits, and scanning speed. To obtain a high-quality fundus image, the backscattered retinal signal must be efficiently collected, the illumination must meet safety standards, and the scanning speed must be maximized to minimize the effects of eye movement. Fig. S1 illustrates the relationship between the design constraints and the associated trade-offs. To optimize the retinal signal strength, key considerations include: 1) using a longer exposure time until camera saturation is achieved; 2) employing a high-power source to enhance illumination; 3) minimizing the number of pixels on the camera sensor to reduce signal crosstalk; and 4) utilizing a narrow slit with a corresponding pixel count to improve illumination efficiency. Conversely, to meet safety limits, it is essential to reduce both the exposure time and illuminating power. For optimal scanning speed, a higher pixel count per acquisition is preferred, as this minimizes the number of frames required for a complete image. Accordingly, a wider slit would be necessary to accommodate a larger number of pixels per frame.

S2: System Characterization

**Fig. S2**. (A) The model eye used for FOV quantification follows the eye angle. (B) The acquired artificial fundus image of the model eye. The fourth ring represents an eye angle of 80˚, which is a close approximation of a 60˚ external angle. (C) The quantified system resolution with a central-installed USAF 1951 resolution target.

The developed LS-CFC device was benchmarked for its imaging performance. The model eye (Rowe Technical Design, Inc., USA) used for FOV quantification follows the eye angle shown in Fig. S2 (A). The fourth ring represents an 80˚ eye angle; hence, a 60˚ external angle slightly extends over the fourth ring.

A model eye incorporating a centrally positioned 1951 USAF resolution target was used to quantify the system resolution at the retinal plane. Based on the measured pupil-plane illumination beam diameter of approximately 3.5 mm, the ideal diffraction-limited retinal resolution was estimated using the Rayleigh criterion. Assuming a representative visible wavelength of 550 nm and an emmetropic-eye focal length of 17 mm, the theoretical lateral resolution is approximately 3.3 µm, corresponding to approximately 150 lp/mm. This value represents an ideal upper limit based on the effective pupil aperture rather than the expected resolution of the complete imaging system. Based on visual inspection of the USAF 1951 target, the maximum resolvable spatial frequency was estimated to lie between Group 4, Elements 4 and 6, corresponding to approximately 23–28 lp/mm, as shown in Fig. S2(C).

The discrepancy between the theoretical and measured resolution is primarily attributed to aberrations and sampling limitations in the complete optical system. The present design corrects defocus using a tunable lens but does not compensate for higher-order aberrations, and the tunable lens itself may introduce additional aberrations under imperfect alignment. The optical properties of the model eye are also not fully characterized. Together with focus sensitivity and finite detector and scanning sampling, these factors broaden the system point spread function and limit the experimentally achievable resolution. Increasing the sampling density alone therefore would not recover the ideal diffraction-limited resolution.

While the LS-CFC system does not aim to achieve the diffraction-limited resolution of adaptive optics scanning laser ophthalmoscopes, the achieved FOV and resolution are appropriate for its intended role as a low-cost and easily deployable retinal imaging platform. Future work will focus on improving resolution through the incorporation of aberration-compensated optical design, optimization of pupil conjugation and alignment, and evaluation using standardized ISO 10940 methodologies to enable direct comparison with clinical fundus imaging systems.

S3: Image Formation and Corrections

**Fig. S3.** Image formation and correction for line-shaped illumination. **(A)** Upsampled strip image showing the detected centerline, polynomial fit, and corrected result. **(B)** Schematic of strip extraction, realignment, and montage. **(C)** Normalized red, green, and blue intensity distributions along the line direction.

The LS-CFC fundus image is formed by montaging multiple backscattered retinal strips. Each 36 × 1600-pixel strip is acquired before the x-axis galvo advances to the next position, providing sufficient margin for centerline estimation and correction of illumination-line curvature before extraction`	. The central 12 pixels of each corrected strip are then selected to form the final image. The 120 µm illumination slit is projected onto the retina with a line width of approximately 163 µm and subsequently relayed to the camera sensor with a magnification of approximately 0.42, corresponding to a projected line width of approximately 68.5 µm. This closely matches the 70.3 µm width represented by 12 camera pixels, providing a physical basis for the selected detection width. The final choice of 12 pixels was also determined empirically as a compromise between confocal rejection, retinal signal strength, and acquisition speed. Selecting fewer central pixels provides tighter confocal gating and higher contrast but reduces the collected signal and requires more scanning positions to cover the same field of view, thereby increasing the acquisition time. After acquiring 120 strip images, the galvo scanner steers the beam vertically to the subsequent starting point and scans the next region. Three vertically offset scan regions are acquired in this manner and subsequently montaged to produce the final wide-field fundus image using PTGui (New House Internet Services B.V., Netherlands).

An exposure time of 1 ms and camera gain of 12 were used, resulting in a total acquisition time of 0.36 s. The short exposure minimized the pupillary light reflex, which typically occurs within ~0.5 s. The achievable acquisition speed was limited by the camera frame rate and the illumination power available within the retinal safety limits. The selected parameters therefore represented an empirical balance between retinal signal strength, image contrast, acquisition time, and susceptibility to eye motion.

The system-level artifacts were addressed too. As shown in Fig. S3 (A), a piece of white paper was imaged to visualize system artifacts. The Gaussian illumination profile causes intensity variation across each strip, producing visible montage seams that can be partly reduced by edge-intensity correction. More uniform illumination, such as a multi-chip LED source, could further mitigate this artifact. In addition, lens distortion caused the illumination line to curve by 1–3 pixels from the center to the edges. We corrected this by fitting the line, selecting pixels along the fitted curve, and realigning them into undistorted strips, as shown in Fig. S3 (B). Lastly, Fig. S3 (C) shows the averaged intensity distribution of the beam in the red, green, and blue channels. The weight of the red channel was corrected by using a ColorChecker (X-Rite Inc., Grand Rapids, United States). The reflectance of each color patch was then verified to ensure that their recorded intensities were consistent across the camera.
